# A Novel Haptic Interactive Approach to Simulation of Surgery Cutting Based on Mesh and Meshless Models

**DOI:** 10.1155/2018/9204949

**Published:** 2018-04-15

**Authors:** Qiangqiang Cheng, Peter X. Liu, Pinhua Lai, Shaoping Xu, Yanni Zou

**Affiliations:** ^1^School of Measuring and Optical Engineering, Nanchang Hangkong University, Nanchang, China; ^2^School of Information Engineering and Mechatronics Engineering, Nanchang University, Nanchang, China; ^3^Department of Systems and Computer Engineering, Carleton University, Ottawa, ON, Canada K1S 5B6; ^4^School of Information Engineering, Nanchang University, Nanchang, China

## Abstract

In the present work, the majority of implemented virtual surgery simulation systems have been based on either a mesh or meshless strategy with regard to soft tissue modelling. To take full advantage of the mesh and meshless models, a novel coupled soft tissue cutting model is proposed. Specifically, the reconstructed virtual soft tissue consists of two essential components. One is associated with surface mesh that is convenient for surface rendering and the other with internal meshless point elements that is used to calculate the force feedback during cutting. To combine two components in a seamless way, virtual points are introduced. During the simulation of cutting, the Bezier curve is used to characterize smooth and vivid incision on the surface mesh. At the same time, the deformation of internal soft tissue caused by cutting operation can be treated as displacements of the internal point elements. Furthermore, we discussed and proved the stability and convergence of the proposed approach theoretically. The real biomechanical tests verified the validity of the introduced model. And the simulation experiments show that the proposed approach offers high computational efficiency and good visual effect, enabling cutting of soft tissue with high stability.

## 1. Introduction

With the rapid development of computer 3D processing capacity and the emergence of low-cost sensors, major advances have been achieved in the past decades in the areas of virtual reality (VR) [1, 2]. As an important VR application field, virtual surgery and trials present a safe and potentially effective method by testing on virtual patients [3, 4]. Moreover, surgical robot has rapidly expanded in medicine by reducing the size, weight, complexity, and costs of robotic surgery systems [5]. Surgery typically involves pressing, cutting, burning, tearing, suction, and other conventional surgical procedures. As the most frequently used operation, the soft tissue surgical cutting is indispensable in the virtual surgery system. However, the soft tissue is a special composite elastic material with complex material properties such as anisotropy, inhomogeneous viscoelasticity, and approximate incompressibility. In [6, 7], we had successfully simulated the deformation of soft tissue regarding nonlinear viscoelasticity. Nevertheless, simulating soft tissue cutting is significantly different from the deformation, for the dramatic changes of the geometry and topology during cutting.

In the last decade, researchers have proposed a number of soft tissue cutting models. In [8], we reviewed the existing cutting models and summarized the advantages and disadvantages of the various modelling methods. According to whether virtual organ objects need to be premeshed or not during the simulation process, the soft tissue cutting models can be roughly divided into two modelling architectures: mesh-based approaches and meshless-based approaches. The element removal method proposed by Nielsen [9] was the earliest cutting simulation method based on mesh. In order to achieve more realistic effect, Nielsen and other scholars proposed some improved models, such as the vertex split method [10], the vertex duplication method [11], and the surface constraint method [12]. Furthermore, to express the internal change of soft tissue during cutting, Bielser et al. proposed a voxel subdivision method [13] that splits the objects by using the volumetric element tetrahedron. To solve the contradiction between calculation speed and accuracy of simulation, the improved models such as the virtual node method [14, 15], adaptive method [16], and the $\sqrt{3}$ subdivision method [17] were proposed successively. Recently, the extended finite element method (XFEM) used in researching metal crack was introduced into the soft tissue cutting simulation [18–21].

The meshless method was introduced into the computer graphics by Desbrun and Cani in 1995 firstly [22]. Subsequently, Müller et al. proposed a meshless method based on the displacement vector field gradient to simulate elastic, plastic, and insoluble objects [23]. Pauly et al. used the transparent guidelines to deal with discontinuity caused by the cutting and fracture on the basis of this algorithm and realized the animation of the brittle solids, highly plastic soft cutting, and fracture [24]. In 2012, Jung et al. proposed a topology fast subdivision meshless cutting algorithm which was based on undirected graph and bounding volume hierarchy (BVH). The results show that this algorithm has fast calculation speed and high efficiency and can meet real-time requirements [25]. These meshless methods are based on the point elements, and the shape function does not participate in the discretization and calculation process. In 2010, Joldes et al. firstly proposed meshless total Lagrangian adaptive dynamic relaxation method (MTLADR), which belongs to a kind of classical meshless method, that is, element-free Galerkin (EFG) [26]. Based on the MTLADR, in 2012, Jin et al. proposed a cutting algorithm adopting a level set technique and achieved a realistic cutting simulation of 2D objects [27]. In 2013, the research group successfully extended this work to 3D objects [28]. Unlike the EFG method, the radial point interpolation meshless method (RPIM) does not need any integration cell and is applied to solve telegraph and heat diffusion equations and time fractional diffusion-wave equation [29, 30]. Recently, Zou et al. simulated the deformation of soft tissue based on RPIM [31].

The mesh-based models had solid theoretical basis, mature algorithms, and many research institutes had published the related open source code, such as chai3D [32] (developed by Stanford University) and the SOFA [33] (developed by INRIA). However, no matter what kind of cutting models, they need pretreatment of mesh. Updating and reconstructing the mesh in the process of cutting may easily generate some narrow and distorted mesh that causes the instability of the soft tissue cutting simulation system. Moreover, it requires a lot of computational costs to maintain this mesh relationship during the process of cutting. By contrast, the meshless-based soft tissue cutting model reconstructs the virtual tissue on the basis of the discrete and separates point elements and the relationship among each point element does not associate with the mesh. It does not need special maintenance for the topological structure of point elements. Therefore, this will eliminate the possibility of generating the distorted mesh, improve the stability of the model, and reduce the computational burden of the algorithm. However, it is difficult to exert the boundary conditions when analyzing the relationships between stress and strain of the elements. Therefore, in order to take full advantage of the characteristics of these two strategies and avoid their shortcomings, many researchers combined the two strategies and established a coupled cutting model based on the mesh and meshless methods. In 2012, inspired by this idea, Zhou et al. argued that the cutting model should be established by a group of surface patches showing the outline of objects and a set of points describing the internal structure of the model [34]. However, since the texture coordinates of the inserted internal vertex can not be obtained, there is no texture in the new cutting surface. Subsequently, Peng et al. combined the vertex duplication method with the body element transfer chain deformation model to realize simulation of soft tissue cutting [35]. In order to reflect the diversity of the components of the soft tissue and show physical properties of the soft tissue more accurately, the researchers utilized a coupled approach to simulate deformation and cutting of anisotropic objects [36–38].

The above coupled methods are promising, but the integrity and computational efficiency of these methods are not well considered. As mentioned above, the coupled method handles the internal and surface regions of soft tissue with different models. So, it is easy to find perceptible artifacts at the boundaries of the two models. This will undermine the integrity of the model and visual effect of cutting simulation. In addition, due to the complex data structure and process, the computational burden is also heavy. To address these problems, we proposed a novel coupled model of the soft tissue cutting model based on mesh and meshless approaches for surgical simulation. Specifically, we introduce virtual points into the coupled model to combine the surface mesh model and the internal meshless model together. The virtual points are obtained by interpolating through points which is located on the cutting boundary that is determined by the surface mesh and cutting path. Moreover, the level set method is used to quickly trace the cutting path and divide the internal points into two regions, that is, the cutting-affected region and cutting-unaffected region. The displacements of the internal point elements in the cutting-unaffected region do not need calculating. On the contrary, the displacements of the internal point elements in the cutting-affected region will be calculated under the framework of meshless. The way of utilizing the virtual points means that the deformations caused by the cutting will be propagated to the internal points. In this way, the two different models are seamlessly combined together. Consequently, proper use of this strategy can improve the computational efficiency. Furthermore, the force feedback is more precise by using an oscillating function. Experimental and comparison results show that the force feedback calculated from our model and those from biomechanical tests is consistent with each other. The maximum relative error is less than 6.2%. The stability and convergence of our approach were discussed and theoretically proven. Finally, we applied the proposed model to a neurosurgery simulation system to demonstrate its applicability.

The remainder of this paper is organized as follows. In the next section, we briefly introduce the integrated framework of our approach. We propose the algorithm based on Bezier curve for surface mesh incision and internal point element deformation based on meshless in Section 3 and Section 4, respectively. In Section 5, the virtual points are introduced to combine the surface mesh model and the internal meshless model. Numerical experiments and implementation are shown in Section 6. Finally, conclusions and some suggestions for future work are given in Section 7.

## 2. The Framework of the Proposed Coupled Approach

Actually, the real cutting process of soft tissue can be divided into two parts: surface cutting and internal cutting. Therefore, we describe the surface and the interior of soft tissue cutting simulation. We utilize Bezier curve to characterize surface incision. Bezier curve can characterize the surface incision quickly and easily, because it has some fine property, such as easy constructing and simple controlling. During the cutting, the soft tissue is split along the cutting plane in both directions. It is reasonable to assume that there exist equal and opposite forces perpendicular to the cut plane. Therefore, the internal cutting of the soft tissue is treated as deformation under the influence of the scalpel, and the internal point elements of soft tissue are generally deformed under these forces. As such, our approach avoids the defect of the mesh-based approach that is generally difficult to maintain topology relationships of vertexes and system stability, and it solves the difficult problem of exerting boundary conditions and reduces the execution time significantly in the meshless-based approaches.

The main steps of our coupled approach are described as follows:

Step 1. Reconstruct the virtual model of soft tissue. The model includes surface mesh and internal point elements.
Step 2. Load the two parts of the model.
Step 3.1. Surface part
  (a) get the cutting path according to the collision detection;
  (b) determine the influence domain of cutting;
  (c) determine the start, end, and control points of cutting;
  (d) construct the incision with the Bezier curve.
Step 3.2. Internal part
  (a) let *φ* denote the scalpel surface, which is the cutting plane formed by the cutting path obtained from collision detection; let *ϕ* denote the vertical plane which is perpendicular to the plane of the scalpel surface;
  (b) utilize the level set method to track the cutting path and classify the point elements into two kinds: cutting-affected point elements and cutting-unaffected point elements;
  (c) calculate the deformation displacement of point elements affected by the cutting region;
  (d) calculate the new coordinates of all of the point elements.
Step 4. Render the virtual model of soft tissue.

**Figure 1:**
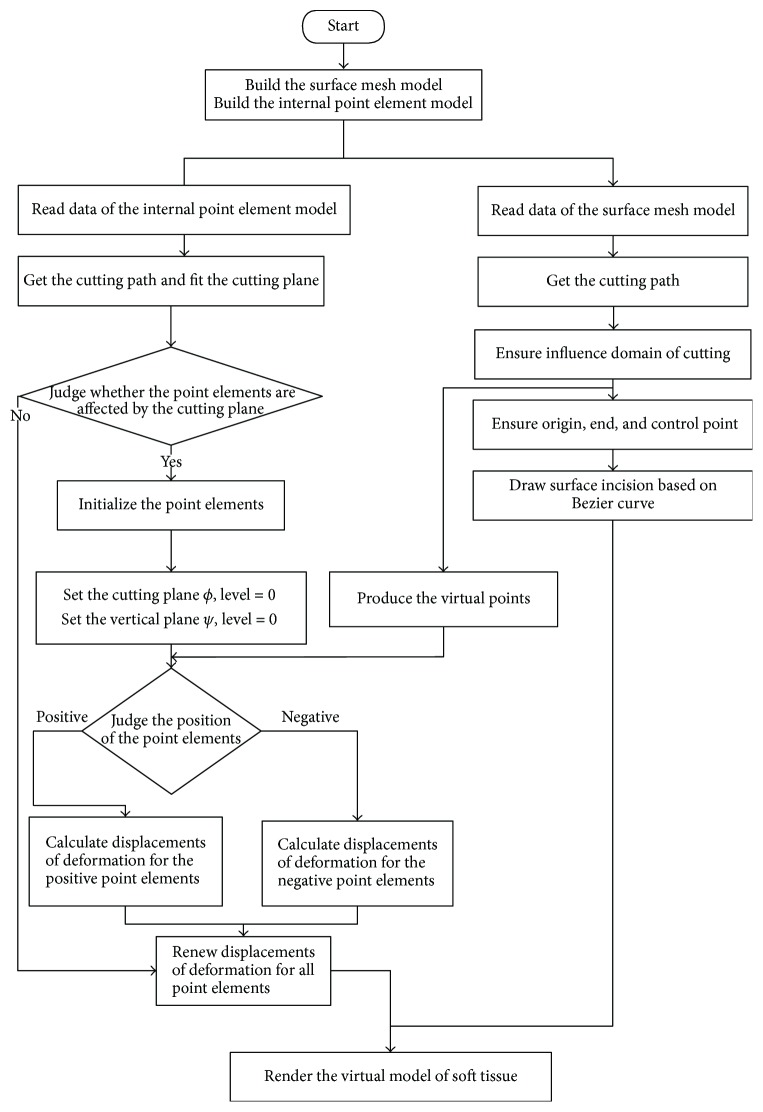
Flow chart of the coupled cutting model.

The entire simulation of the soft tissue cutting process flow is shown in Figure 1.

As such, since the virtual points produced by surface mesh can be treated as boundary points, the coupled cutting model proposed by us solved the problem of imposing the essential boundary condition in meshless. Furthermore, with the introduction of the lever set method, the internal point elements have subdivided into cutting affected where displacements need updating and cutting unaffected where displacements do not need updating. Therefore, the computing speed of the coupled model is accelerated. Moreover, unlike the traditional mesh-based cutting method, our coupled model uses some discrete points to character the deformation of the soft tissue caused by cutting. So, the problem of system instability is avoided. In short, assuring visual effect, the coupled model can maintain high and stable calculating speed.

## 3. Modelling Surface Incision

Generally, spline curve falls into two categories: interpolation spline and approximation spline. Interpolation spline is typically used for the digital drawing or animation design, while approximation spline is generally used to construct the surface of the object. Bezier curve belongs to approximation spline, which is a free curve that does not need the usage of a standard algebraic equation to describe, as long as the control points are given. In this work, when users perform cutting operations with the scalpel, the cutting edge is characterized by Bezier curve. Moreover, Bezier curve always starts with the first control points and ends with the last control points; so, it can truly reflect the cutting length.

Given a set of control points *P*_*i*_, Bezier curve is defined as

(1) $$\begin{array}{c} P\left(t\right)=\overset{n}{\underset{i=0}{\sum }}P_{i}B_{i,n}\left(t\right),\;t\in \left[0,1\right], \end{array}$$

where the parameter *t* ∈ [0, 1], *n* is the number of control points, and *B*_*i*,*n*_(*t*) is the Bernstein polynomial, which is called the *n*th Bernstein basis function. It is defined as

(2) $$\begin{array}{c} B_{i,n}\left(t\right)=C_{n}^{i}t^{i}{\left(1-t\right)}^{n-1}\;\left(i=0,1,\ldots ,n\right),\\ C_{n}^{i}=\frac{n!}{i!\left(n-i\right)!}. \end{array}$$

In order to reduce the amount of calculation and accelerate the speed of incision rendering, we select the quadratic Bezier curve to achieve the reproduction of the incision. The quadratic Bezier curve equation is defined as

(3) $$\begin{array}{c} P\left(t\right)=\overset{2}{\underset{n=0}{\sum }}P_{i}B_{i,2}\left(t\right)={\left(1-t\right)}^{2}P_{0}+2t\left(1-t\right)P_{1}+t^{2}P_{2}=\left(P_{2}-2P_{1}+P_{0}\right)t^{2}+2\left(P_{1}-P_{0}\right)t+P_{0},\;t\in \left[0,1\right]. \end{array}$$

**Figure 2:**
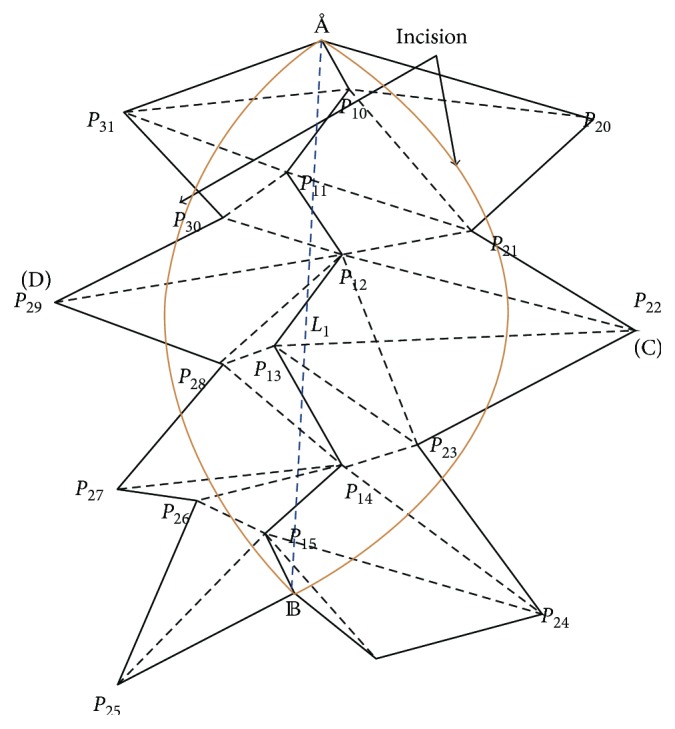
Surface mesh incision.

As shown in Figure 2, the detail steps are described as follows.

**Step 1 .**

Get the surface vertexes of the cutting path according to the results of collision detection, denoted as *P*1 = {*P*_10_, *P*_11_, *P*_12_, *P*_13_, *P*_14_, *P*_15_}.

**Step 2 .**

Search the triangular meshes which the above vertexes belong to, that is, the region affected by the cutting, which needs restructuring the mesh, and mark all the vertices contained in the affected meshes, denoted as *P*2 = {*P*_20_, *P*_21_, *P*_22_, *P*_23_, *P*_24_, *P*_25_, *P*_26_, *P*_27_, *P*_28_, *P*_29_, *P*_30_, *P*_31_}.

**Step 3 .**

Connect the straight line *L*1 between the start point A and end point B and all of the vertexes in point set *P*2 distributed on both sides of *L*1, marking the points on the left as positive vertexes and on the right as negative vertexes.

**Step 4 .**

Traverse all of the vertexes in point set *P*2 and calculate the distances between these vertexes and *L*1, and then, select the two points (C and D) which have the maximum distances to *L*1 in the positive and negative point sets, respectively.

**Step 5 .**

Construct two quadratic Bezier curves to represent incision. One Bezier curve is determined by vertexes A, B, and C, and the other one is determined by vertexes A, B, and D.

## 4. Deformation of Internal Point Elements

In the mesh method, all of the vertexes are connected by the mesh. The mesh includes topology-related information, such as vertexes, edges, and triangles. As the connection among each vertex is fixed, so, the search process can be carried out efficiently. However, maintaining such mesh data structure requires a lot of memory and a very large amount of calculation, especially during the cutting process. Currently, how to maintain a fine mesh structure for the complex models subjected to cutting is one of the challenges in computer graphics. Cutting operation would certainly destroy the initial mesh structure, and it needs to be remeshed. Moreover, it is prone to appear narrow and sharp distortion mesh in this process, resulting in the instability of the simulation system. Compared with the conventional mesh method, the meshless method is more suitable for simulating large deformation and cutting of soft tissue. In the meshless method, the object is discretized by a series of independent point elements and does not need to establish mesh structure. Therefore, it avoids reconstructing the mesh after large deformation and cutting.

### 4.1. Virtual Scalpel

**Figure 3:**
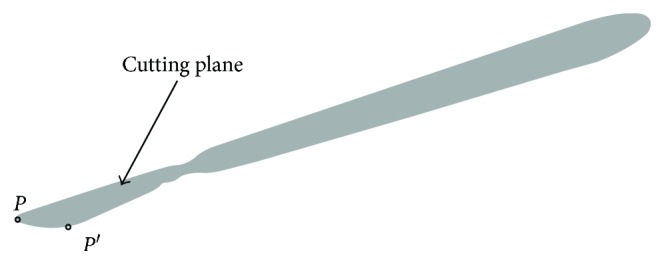
Virtual scalpel.

The first step of simulating the cutting process of soft tissue is to determine the cutting path, which is the intersecting line between the scalpel and soft tissue surface. In order to obtain the cutting path, we need to create a virtual scalpel and obtain a cutting plane. The conventional method represents the scalpel as having three virtual points that can be used to define a plane. However, this method requires constantly updating the position of three virtual points, which needs a large amount of calculation. Therefore, we propose a simple method which can quickly obtain the cutting plane. In our work, the scalpel is only represented by point *P* at the tip of the tool and the other points of the tool are only used for rendering, because they do not participate in calculating the subsequent collision detection and obtaining the blade surface. As we know, during the process of a real operation, the doctors always keep their eyes perpendicular to the soft tissue when cutting. Thus, we assume that the blade surface is always perpendicular to the surface of soft tissue. In order to represent the cutting plane, we add another point *P*′ whose position is provided and updated by a force feedback device. As such, the cutting plane can be determined by the two points and a vertical direction, as shown in Figure 3.

### 4.2. Tracking the Cutting Path

During the cutting, the position of the cutting plane changes with the cutting path; so, how to track the cutting path quickly and effectively is the key to detecting the position of cutting. In this work, the level set method is adopted to track cutting interfaces and shapes. The basic idea of the level set is to consider the boundary surface as the zero level set of a function *ψ* (called the level set function) in the higher dimension space, and the speed of the boundary surface also expands to the level set function in higher dimension. As such, we can derive the developmental equation meeting the condition of the level set function. We can find the zero level set of the new level set function by solving this equation and evolving the level set function. It is easy to track the topology structure changes of the objects by this way. Hereby, we adopt the level set method to track the cutting path. The detailed steps are described as follows.

Use the level set function in higher dimension to represent the cutting path *γ*(*t*), that is,

(4) $$\begin{array}{c} \gamma \left(t\right)=\left\{x\in R^{2}:\varphi \left(X,t\right)=0\right\}, \end{array}$$

where *X* = (*x*, *y*, *z*) is the coordinate of point element, *t* is the time, and *φ*(*X*, *t*) is the level function with time.

The cutting path can be divided into two subdomains, and the movement of *γ*(*t*) can be obtained by the level evolution equation as

(5) $$\begin{array}{c} \varphi_{t}+v\left\|\nabla_{\varphi }\right\|=0,\\ \varphi \left(X,t=0\right)=constant, \end{array}$$

where *φ*_*t*_ is the derivative of the level set function, *v* is the velocity function, and *∇*_*φ*_ is the gradient of the level set function.

**Figure 4:**
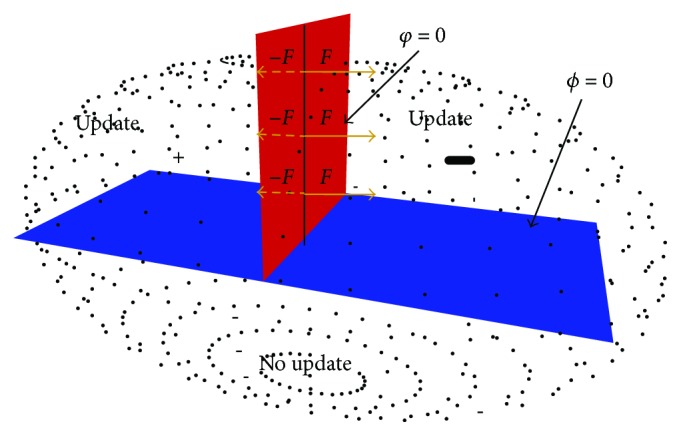
Tracking the cutting path based on the level set method.

As shown in Figure 4, the blade and the vertical plane of the blade surface are set by the level set of two level 0s, determined by the functions *φ* and *ϕ*, respectively.

(6) $$\begin{array}{c} \varphi \left(f\left(X,X_{i}\right),t=0\right)=0,\\ \phi \left(f\left(X,X_{i}\right),t=0\right)=0, \end{array}$$

where *f*(*X*, *X*_*i*_) is the spatial distance of point elements *X* and *X*_*i*_. *X* is a given point element and *X*_*i*_ is the point element on level 0.

The initial state of the level set functions *φ* and *ϕ* is defined as (7) and (8), respectively.

(7) $$\begin{array}{c} \varphi \left(x,t\right)=\pm \min\left(\left\|X-X_{c}\right\|\right), \end{array}$$

(8) $$\begin{array}{c} \phi \left(x,t\right)=\pm \min\left(\left\|X-X_{v}\right\|\right). \end{array}$$

Among them, *X*_*c*_ is the point element on the cutting path and *X*_*v*_ is the point element on the plane which is perpendicular to the blade. *φ* and *ϕ* are the signed distance functions of cutting path *γ*(*t*), that is, the values of *φ* and *ϕ* are the shortest distance between the point element to the blade plane and the vertical plane, and their signs depend on the point element location.

The vertical plane divides the whole internal point model into upper and lower parts (see Figure 4). The upper part of the point elements affected by the cutting (blade surface) will be displaced and need updating. The lower parts of the point elements are not affected by the cutting (the displacement is zero) and need no updating. The point elements located above the vertical plane are divided into the left and right parts by the blade surface; the point elements of the left part are marked “+” and ones of right part are marked “−.” The two parts of the point elements will move to the left and right directions, respectively, during the cutting process. As such, the calculating speed of the coupled model increases greatly by using the level set method.

### 4.3. Cutting Deformation

In our work, soft tissue cutting can be simply divided into surface split and internal deformation. After the soft tissue has been split, the point elements of the left and right sides are affected by two forces which result in deformation. Müller et al. proposed the moving least square approximation method (MLS) based on displacement vector field gradient to simulate the deformation of the elastomer [23]. Unlike other meshless methods, this method does not have special preprocessing steps such as establishing background integration mesh for calculating the displacement of the point elements in the EFG method. The new displacements of the point elements after deformation can be obtained by calculating the displacement vector field gradient of each point element with a much faster implementation, avoiding the complicated evaluation of integrals. This paper adopts the method to calculate the deformation displacements of point elements, which are influenced by cutting.

Assuming a Hookean material, the stress *σ* and strain *ε* of soft tissue are linearly related as

(9) $$\begin{array}{c} \sigma_{i}=E\varepsilon_{i}, \end{array}$$

where *E* is the material parameter.

Then, the Green's nonlinear strain tensor in computer graphics can be defined as

(10) $$\begin{array}{c} \varepsilon_{i}=\frac{1}{2}\left(\nabla_{U_{i}}+{\left[\nabla_{U_{i}}\right]}^{T}+\nabla_{U_{i}}\nabla_{U_{i}}\right), \end{array}$$

where *∇*_*U*_*i*__ = (*∇*_*u*_*i*__, *∇*_*v*_*i*__, *∇*_*w*_*i*__) is the displacement vector gradient of three directions of each point element defined as

(11) $$\begin{array}{c} \nabla_{u_{i}}=M^{-1}\left(\sum_{j}\left(u_{j}-u_{i}\right)x_{ij}w_{ij}\right),\\ \nabla_{v_{i}}=M^{-1}\left(\sum_{j}\left(v_{j}-v_{i}\right)x_{ij}w_{ij}\right),\\ \nabla_{w_{i}}=M^{-1}\left(\sum_{j}\left(w_{j}-w_{i}\right)x_{ij}w_{ij}\right),\\ M^{-1}=\left(\sum_{j}x_{ij}x_{ij}^{T}w_{ij}\right). \end{array}$$

Among *x*_*ij*_ = *x*_*i*_ − *x*_*j*_, *x*_*i*_ represents the coordinates of the point elements to be calculated and *x*_*j*_ represents point elements in the influence domain of calculating point elements. *ω*_*ij*_ is the weight and *u*_*i*_, *v*_*i*_, *w*_*i*_ and *u*_*j*_, *v*_*j*_, *w*_*j*_ are the displacement of given point elements and point elements in the influence domain of calculating point elements, respectively.

As such, the physical strength *f* can be computed as

(12) $$\begin{array}{c} f_{j}=f_{j}+B_{i}\left(x_{ij}\omega_{ij}\right), \end{array}$$

(13) $$\begin{array}{c} f_{i}=f_{i}-B_{i}\left(x_{ij}\omega_{ij}\right), \end{array}$$

(14) $$\begin{array}{c} B_{i}=-2v_{i}\left(\nabla_{U_{i}}+I\right)\sigma_{i}M^{-1}, \end{array}$$

where *v*_*i*_ is the volume of the given point elements, *∇*_*U*_*i*__ is the displacement gradient, and **I** is an identity matrix.

Then, the new displacement of the point elements can be derived as

(15) $$\begin{array}{c} {\dot{U}}_{i}={\dot{U}}_{i}+\Delta t\frac{f_{i}}{m_{i}},\\ U_{i}=U_{i}+\Delta t{\dot{U}}_{i}, \end{array}$$

where *U*_*i*_ = *U*(*u*(*x*, *y*, *z*), *v*(*x*, *y*, *z*), *w*(*x*, *y*, *z*)) is the displacement of the given point element after cutting. Δ*t* is the time step and *m*_*i*_ is the quality of the given point element.

Finally, the new position coordinates of the point elements can be calculated as

(16) $$\begin{array}{c} x_{i}=x_{i}+u_{i}\\ y_{i}=y_{i}+v_{i}\\ z_{i}=z_{i}+w_{i}. \end{array}$$

Therefore, we can calculate the stress, strain, and physical strength of point elements through the displacement vector gradient of point elements and then utilize the basic laws of kinematics to obtain the displacements caused by the deformation of point elements. Since we use the displacement of discrete points to characterize the deformation of soft tissue, the instability problems can be avoided.

According to biomechanical test, although there is a downtrending for the cutting force, it vibrated around a particular value. Since the force calculated by our model decreases monotonously, it may not characterize the real situation accurately. Therefore, we modify the force model with an oscillating function.

(17) $$\begin{array}{c} \Gamma \left(t,d\right)=e^{-at}\sin\left(bt\right)\Upsilon \left(d\right), \end{array}$$

where *t* is the time step, *d* is the depth of cutting, Υ(*d*) is the linear function with *d*, and *a* and *b* are two coefficients that can be determined from the experimental data.

Multiplying (13) by Γ(*t*, *d*), the force feedback exerted for point *i* can be calculated by

(18) $$\begin{array}{c} f_{i-feedback}=\left(f_{i}-B_{i}\left(x_{ij}\omega_{ij}\right)\right)\Gamma \left(t,d\right). \end{array}$$

**Figure 5:**
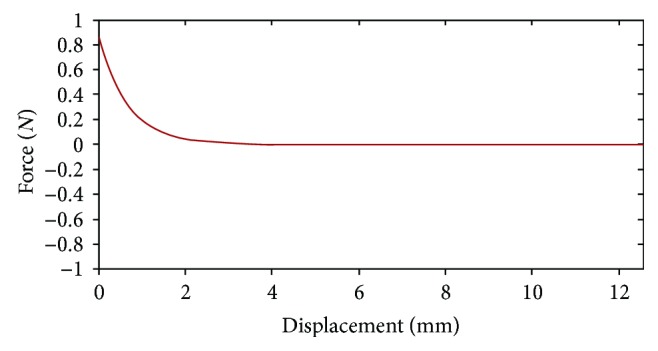
The force versus displacement curve for a soft tissue before adding oscillating function.

**Figure 6:**
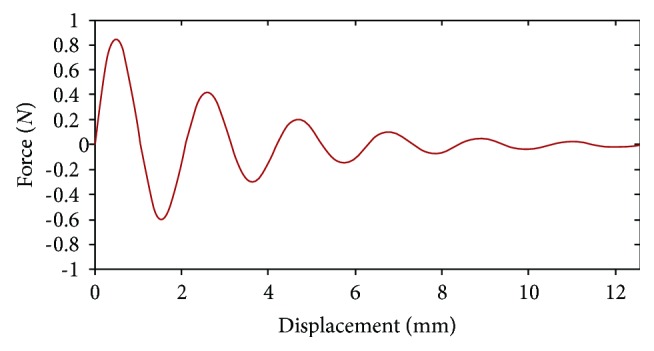
The modified force feedback versus displacement.

Figure 5 is the force versus displacement curve for a soft tissue before adding oscillating function. The modified force feedback versus displacement is shown in Figure 6. By comparing Figures 5 and 6, the modified force feedback exhibits a matching behavior to the real cutting which varied in small scale.

## 5. Coupled Soft Tissue Model

**Figure 7:**
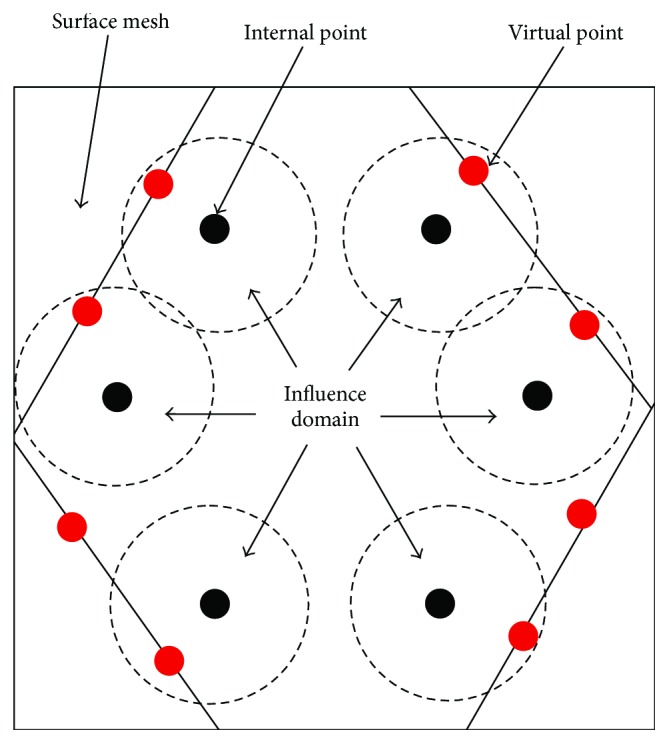
A definition of virtual point in a coupled model.

The surface cutting model and internal point element deformation model based on mesh and meshless, respectively, which have been described in the previous sections, are independent of each other. So, they can not directly be used to simulate the soft tissue cutting without appropriate modification. In fact, the deformation of internal point elements is closely related to the incision of surface mesh. Precisely, the boundary of incision determines the displacement of the internal point elements. Therefore, in order to establish a relationship between these two models, we add virtual points to the incision boundary of surface mesh that construct the incision as shown in Figure 7. We obtain the virtual points by interpolation between the vertexes of the boundary mesh (here, we use linear interpolation). When calculating the displacements of the internal point elements, the virtual points are regarded as the common internal points and work together with the internal point elements under the meshless framework. Therefore, the displacements of virtual points resulting from the incision will cause the internal point elements (adjacent to virtual points) to displace their original positions correspondingly. The above-described process is repeated until the predefined maximum search step is reached, resulting in the deformation of internal point elements. In this work, the virtual points are only used to calculate the displacements of internal point elements, but they do not participate in the rendering of surface incision and internal deformation. So, we call them as “virtual” points. Their roles are as follows: on the one hand, they can track the variation of surface incision and can be treated as boundary points and on the other hand, they construct constraint relationship between the two models, and the internal deformation varied with the surface incision. In this manner, the variation of internal deformation synchronizes with the surface cutting incision; the surface mesh model and internal meshless model are seamlessly combined together by the proposed virtual points. Furthermore, it is noted that our virtual point-based approach does not increase the rendering burden of the whole system. Therefore, the challenge of imposing boundary conditions in meshless is solved by introducing virtual points.

## 6. Numerical Results and Algorithm Verification

### 6.1. Numerical Experiment

**Figure 8:**
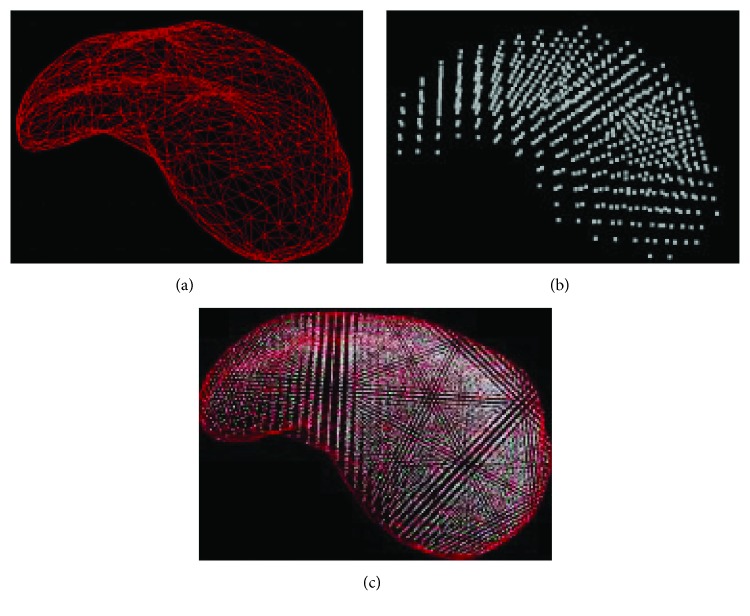
Coupled model reconstruction. (a) Surface mesh reconstructed, (b) internal point element reconstructed, and (c) the integrality reconstructed model.

In order to verify the simulating effect of the proposed approach in this paper, we have carried out the numerical experiments on the human liver and shoulder models. We have obtained a 3D liver mesh model of the soft tissue by using Mimics, which is a commercial medical image controlling software. The reconstructed 3D liver mesh model (length: 250 mm, width: 150 mm, and height: 50 mm) is shown in Figure 8(a). In order to quickly generate the internal point elements of the soft tissue, we have used a subdividing method based on equal distance interpolation. The result of equal distance interpolation subdivision as mentioned above is shown in Figure 8(b).  Figure 8(c) shows the integrality reconstructed model of the liver which includes surface mesh and internal point elements. The virtual human liver model is composed of 508 triangles, and we utilize the fast and efficient subdivision method based on the equidistant interpolation to generate 1526 internal point elements.

**Figure 9:**
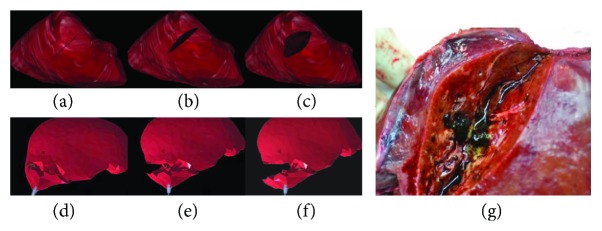
Cutting simulation comparison. (a–c) The liver incision obtained by our method with different cutting depths, (d–f) the liver incision formed by Cotin et al. with different cutting depths, and (g) real incision.

**Figure 10:**
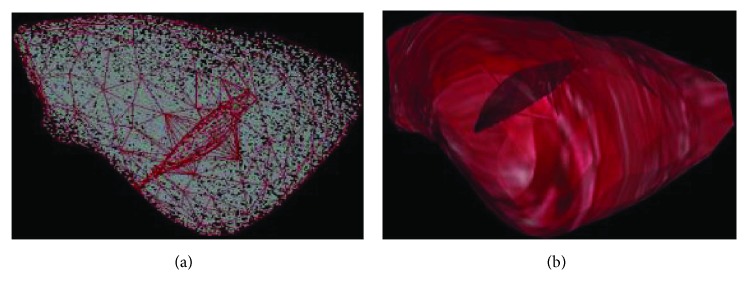
Cutting simulation result. (a) No rendering and (b) with rendering.

The cutting simulation test was carried out on the general PC machine (Intel(R) Core(TM) i7-4510U CPU 2.0 GHz, 2.6 GHz, RAM 7.68 G), and the results are shown in Figure 9. Figures 9(a)–9(c) show the different incisions in the surgery at different depths of cutting. The final size of the incision is as follows: length: 62 mm, width: 45 mm, and height: 42 mm. Figures 9(d)–9(f) are the cutting simulation results proposed by Cotin et al., who coupled the mesh and mass-spring model to simulate the cutting [39]. Figure 9(g) is a real incision of human soft tissue. It can be seen that the Cotin's cutting simulation results are different from the real cutting. The former incision is zigzag, but the latter is smooth. One can observe in Figure 10 that the surface shape of the incision generated by Bezier curve is consistent with the real one. The internal point element deformation which is simulated by the meshless method based on displacement vector gradient can well characterize the internal circumstances of the real incision.

### 6.2. Haptic Rendering of Cutting

**Figure 11:**
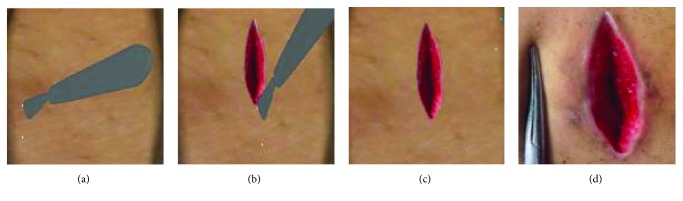
Simulation of the cutting of the human shoulder soft tissue. (a) Starting of cutting, (b) cutting, (c) virtual incision, and (d) real incision.

In addition to visual effect, haptic interaction is also very important for surgical simulators [40]. In order to enhance the reality and immersion of virtual cutting, we add the force feedback in the simulation of soft tissue cutting.  Figure 11 describes a human shoulder soft tissue cutting simulation. It consists of 1842 surface triangles and 4812 internal point elements. The user operates the force feedback device to control a virtual tool in the screen for soft tissue cutting. It can be seen from the comparison between Figures 11(d) and 11(c) that the incision of a virtual operation is smooth and the shape of incision is consistent with the actual surgical incision. Because the number of surface triangles and internal point elements is more than the liver model, the rendering effect of incision is more vivid.

**Figure 12:**
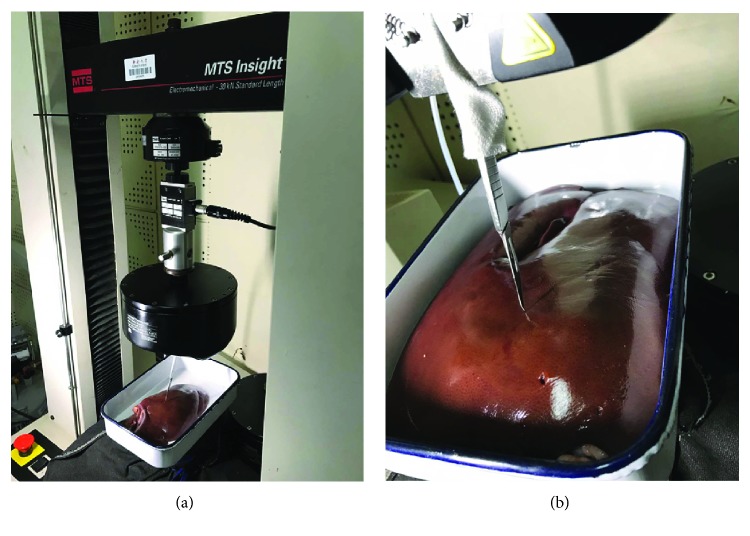
Biomechanical experiment snapshot. (a) Electromechanical universal testing system and (b) porcine liver cutting.

To describe and compare the real biomechanical responses, porcine liver cutting experiments were performed. We use the Insight 30 electromechanical universal testing system that is provided by MTS (Mechanical Testing and Sensing) corporation to perform experiments as shown in Figure 12. The key performance indicators are as follows: (1) speed ranges from 0.001 mm/s to 500 mm/s, (2) velocity resolution 0.005%, (3) displacement resolution 0.0001 mm, (4) force measuring accuracy 0.5% (with force load from 10 N to 10 KN), and (5) displacement precision 0.0001 mm. In our experimental setup, we need to vary the cutting speed from 5 mm/min, 10 mm/min, and 30 mm/min. According to preliminary work, the cutting force that needs measuring ranged from 0 N to 30 N. Therefore, the testing system meets requirements with our experiment.

**Figure 13:**
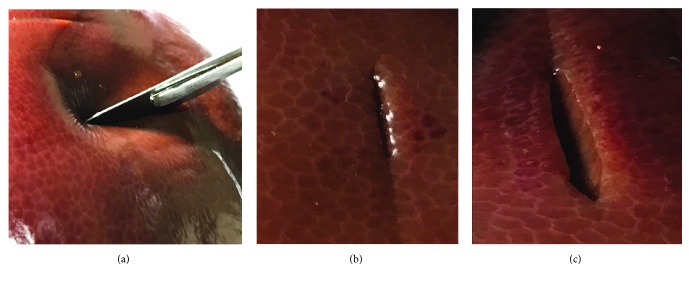
Real cutting process. (a) Scalpel penetrating into soft tissue, (b) small incision, and (c) large incision.

**Figure 14:**
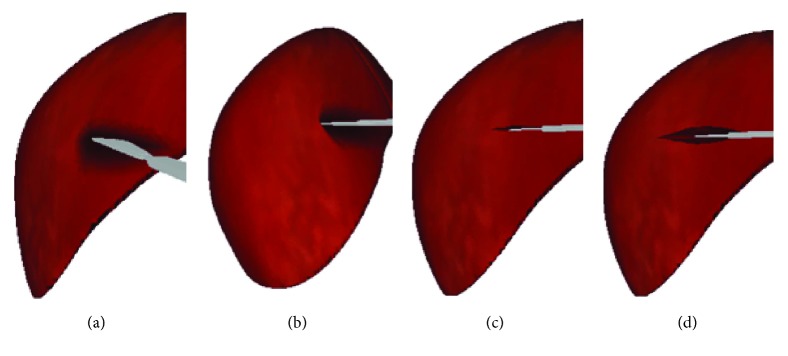
Cutting process simulation. (a-b) Scalpel penetrating into soft tissue, (c) small incision, and (d) large incision.

In general, the properties of soft tissue ex vivo (out of the living) are quite different with in vivo (in the living). To maintain the properties of the liver as similar as possible to the in vivo soft tissue, we selected a fresh porcine liver from a six-month-old pig in a slaughterhouse and transported it to test sites within 2 h post mortem. The experimental process is shown in Figure 13. And the simulated cutting process is shown in Figure 14. By comparing Figures 13 and 14, the simulated results are in good agreement with experimental ones. Figures 14(a) and 14(b) are the simulated results when the scalpel is introduced into the liver from different angles. Throughout this process, the deformation displacement increases with the scalpel penetrating into the liver. And the force applied to the liver increases constantly. When the force applied to the soft tissue exceeds the ultimate force that it can endure, the liver will be cut into two. Once the liver is cut open, it will deform under the scalpel, and the incision will gradually become bigger. It can be seen in Figures 14(c) and 14(d) that the simulated incision of the liver is smooth and vivid. Therefore, the simulated results on different cutting processes show that this method achieves better visual effect.

**Figure 15:**
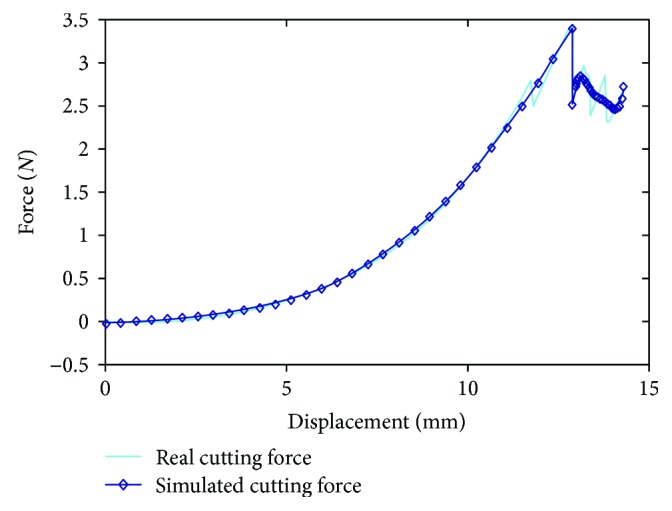
A comparison of the real cutting force curve and simulated cutting curve of our model.

Moreover, our model can be simulated in a high degree of accuracy with the real biomechanical cutting experiment. The relationship curves of displacement and compression force obtained from the experiment are shown in Figure 15. It can be seen in Figure 15 that both curves followed a similar pattern that is composed of three segments. In the first stage, the magnitude of the force that acted on the soft tissue continuously increases as the cutting process progresses. And the deformation of the soft tissue becomes larger. From the point of view of energy, this is the process of energy accumulation where kinetic energy converted to intrinsic energy. When the deformation reaches the maximum—in other words, the maximum force the soft tissue can endure—it will be cut open. Once the imposed force exceeds the maximum force, the soft tissue will be cut into two with a sudden drop of the force. It means that the cutting moved into its second phase. This is the result of a sudden release of intrinsic energy. This energy has gone into fracture energy, cutting the soft tissue open, and the kinetic energy used for deformation after being cut open. Finally, the cutting force fluctuates around an average value with the cutting energy accumulation and release processes. Since our model does not include the squeezing deformation process of the soft tissue before being cut open, a mathematical function has been utilized to fit the feedback force of the first half part of deformation. The fitting formula of function is as follows:

(19) $$\begin{array}{c} f\left(x\right)=a_{1}*e^{-{\left(\left(x-b_{1}\right)/c_{1}\right)}^{2}}+a_{2}*e^{-{\left(\left(x-b_{2}\right)/c_{2}\right)}^{2}}, \end{array}$$

where *a*_1_ = 15.29, *b*_1_ = 3.164, *c*_1_ = 0.2933, *a*_2_ = 27.69, *b*_2_ = 3.288, and *c*_2_ = 1.746.

As can be seen in Figure 15, these two forces show a good consistency with each other. All of the relative error is less than 6.2%. Taking a deep observation, the maximum error lies in the moment when the soft tissue is cut open. Since there are so many influencing factors and all influencing factors can not be considered comprehensively, it is hard to calculate the value of cutting force accurately during the process of converting kinetic energy to intrinsic energy. Furthermore, the relative error becomes smaller along with the cutting. At the soft tissue deformation stage, the fitting function can better reflect the deformation force feedback of soft tissue after being penetrated by the blade. With the continuous deepening of the cutting tool, the force increases nonlinearly. In the cutting stage, the cutting force feedback of the model is consistent with the real cutting force. The cutting force decreases sharply when soft tissue is cut open. And then, the cutting force gradually reduces and tends to be stabilized. Therefore, the model in this paper can better simulate the real cutting process of soft tissue, its incision is smooth and real, and the cutting force feedback is consistent with the real cutting force.

### 6.3. The Stability and Convergence Analysis

Recalling (13), the iterative equation with time can be expressed as

(20) $$\begin{array}{c} f\left(t+\Delta t\right)=f\left(t\right)-B\left(X\omega \right), \end{array}$$

where *X* = *x*_*ij*_ and *ω* = *ω*_*ij*_.

Substituting (10) and (14) into (20) yields

(21) $$\begin{array}{c} f\left(t+\Delta t\right)=f\left(t\right)-Ev\left(\nabla_{U}+I\right)M^{-1}\left(\nabla_{U}+\nabla_{U}^{T}+\nabla_{U}\nabla_{U}^{T}\right)\left(X\omega \right). \end{array}$$

Suppose that *u*_1_ = *u*, *u*_2_ = *v*, and *u*_3_ = *w* and adding the Kronecker delta function property, we have

(22) $$\begin{array}{c} U\left(X\right)=\Phi^{T}\left(X\right)u_{i}=\overset{n}{\underset{i=2}{\sum }}\phi_{i}\left(X\right)u_{i}, \end{array}$$

where the vector **Φ** ^*T*^(*X*) = *ϕ*_1_(*X*)*ϕ*_2_(*X*)*ϕ*_3_(*X*) ⋯ *ϕ*_*n*_(*X*) and *ϕ*_*i*_(*X*) is as follows:

(23) $$\begin{array}{c} \phi_{i}\left(X\right)=\left\{\begin{array}{c} 0\;\text{if }i\neq j,\;j=1,2,\ldots ,n,\\ 1\;\text{if }i=j,\;i,j=1,2,\ldots ,n. \end{array}\right. \end{array}$$

Furthermore, the Kronecker delta function are the partitions of unity

(24) $$\begin{array}{c} \overset{n}{\underset{i=1}{\sum }}\phi_{i}\left(X\right)=1. \end{array}$$

The derivatives of *U*(*X*) are given by

(25) $$\begin{array}{c} U_{l}\left(X\right)=\Phi_{l}\left(X\right), \end{array}$$

where *l* denotes the coordinates *x*, *y*, and *z*. Also, the high derivatives of *U*(*X*) are easily obtained as

(26) $$\begin{array}{c} U_{l}^{s}\left(X\right)=\Phi_{l}^{\left(s\right)T}\left(X\right). \end{array}$$

The time derivative operator using forward difference can be approximated to

(27) $$\begin{array}{c} \frac{\partial f\left(X,k\Delta t\right)}{\partial t}≅\frac{1}{\Delta t}\left(f^{k+1}\left(X\right)-f^{k}\left(X\right)\right). \end{array}$$

By using the Crank-Nicolson method, we can also employ the following approximation:

(28) $$\begin{array}{c} \Delta f\left(X,t\right)≅\frac{1}{2}\left(\Delta f^{k}\left(X\right)+\Delta f^{k+1}\left(X\right)\right), \end{array}$$

where *f*_*k*_(*X*) = *f*(*X*, *k*Δ*t*), Δ*f* = *∂* ^2^ *f*/*∂x* ^2^ + *∂* ^2^ *f*/*∂y* ^2^ + *∂* ^2^ *f*/*∂z* ^2^, and *X* ^*T*^ = (*x*, *y*, *z*).

Then, (28) can be written as

(29) $$\begin{array}{c} \frac{1}{\Delta t}\left(f^{k+1}\left(X\right)-f^{k}\left(X\right)\right)=\frac{1}{2}\left(\Delta f^{k}\left(X\right)+\Delta f^{k+1}\left(X\right)\right). \end{array}$$

Defining *τ* = 1/Δ*t*, (29) can be rewritten as

(30) $$\begin{array}{c} \tau f^{k+1}\left(X\right)-\Delta f^{k+1}\left(X\right)=\tau f^{k}\left(X\right)+\Delta f^{k}\left(X\right). \end{array}$$

Following [30], we utilize the *L* ^2^ Hilbert space to analyze the stability and convergence. The inner product is as follows:

(31) $$\begin{array}{c} \left(v,w\right)=\int_{\Omega }v\left(X\right)w\left(X\right)dX, \end{array}$$

and its induced norm is as follows:

(32) $$\begin{array}{c} {\left\|v\right\|}_{2}={\left[{\left(v,v\right)}_{2}\right]}^{1/2}=\sqrt{\int_{\Omega }v^{2}\left(X\right)dX}. \end{array}$$

Substituting *k* = 0 into (30) yields

(33) $$\begin{array}{c} \tau f^{1}\left(X\right)-\Delta f^{1}\left(X\right)=\tau f^{0}\left(X\right)+\Delta f^{0}\left(X\right). \end{array}$$

Multiplying (33) by *f* ^1^(*X*), we obtain

(34) $$\begin{array}{c} \tau f^{1}\left(X\right)f^{1}\left(X\right)-\Delta f^{1}\left(X\right)f^{1}\left(X\right)=\tau f^{0}\left(X\right)f^{1}\left(X\right)+\Delta f^{0}\left(X\right)f^{1}\left(X\right). \end{array}$$

Integrating (34) into Ω, and the inner product yields

(35) $$\begin{array}{c} \tau {\left\|f^{1}\right\|}_{2}^{2}-{\left(\Delta^{2}f^{1},f^{1}\right)}_{\Omega }=\tau {\left(f^{0},f^{1}\right)}_{\Omega }+{\left(\Delta^{2}f^{0},f^{1}\right)}_{\Omega }. \end{array}$$

Integration by parts, (35) can be derived as

(36) $$\begin{array}{c} \tau {\left\|f^{1}\right\|}_{2}^{2}+{\left(∆f^{1},f^{1}\right)}_{\Omega }=\tau {\left(f^{0},f^{1}\right)}_{\Omega }-{\left(∆f^{0},{∆f}^{1}\right)}_{\Omega }. \end{array}$$

Assuming that *∂f*_*k*_/*∂X* is monotone, we can obtain

(37) $$\begin{array}{c} \tau {\left\|f^{1}\right\|}_{2}^{2}\leq \tau {\left(f^{0},f^{1}\right)}_{\Omega }. \end{array}$$

According to Schwarz inequality, we have

(38) $$\begin{array}{c} \tau {\left\|f^{1}\right\|}_{2}^{2}\leq \tau {\left\|f^{0}\right\|}_{2}^{2}. \end{array}$$

In the same way, we can obtain

(39) $$\begin{array}{c} \tau {\left\|f^{k+1}\right\|}_{2}^{2}\leq \tau {\left\|f^{0}\right\|}_{2}^{2}. \end{array}$$

Therefore, we can safely come to the conclusion that the proposed method can effectively control correlation with good convergence. Furthermore, we prove the stability and convergence of our implementation by evaluating the results with a real biomechanical experiment. Following the conventional finite element method, we choose the root-mean-square error (RMSE) to approximate the error measure. We define the RMSE as follows:

(40) $$\begin{array}{c} \epsilon_{RMSE}=\sqrt{\frac{\sum_{i=1}^{N}{\left(f_{i}-f_{i}^{*}\right)}^{2}}{N}}, \end{array}$$

where *N* is the number of testing (it is set to 10 in this paper), *f* is the calculated results of our model, and *f* ^∗^ is the result of the real biomechanical experiment.

**Figure 16:**
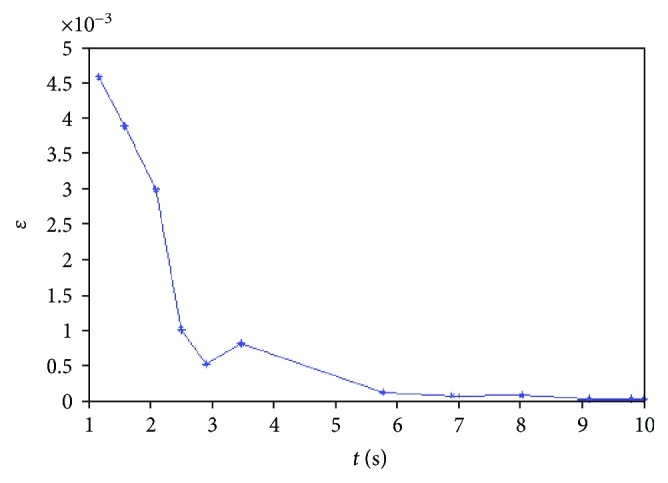
The RMSE of the calculated force and measured from a real experiment.

The RMSE result is shown in Figure 16. As can be seen in Figure 16, the value of RMSE decreases monotonously with respect to time. And the convergence rate is also satisfactory.

### 6.4. The Application of the Virtual Simulation System in Neurosurgery

**Figure 17:**
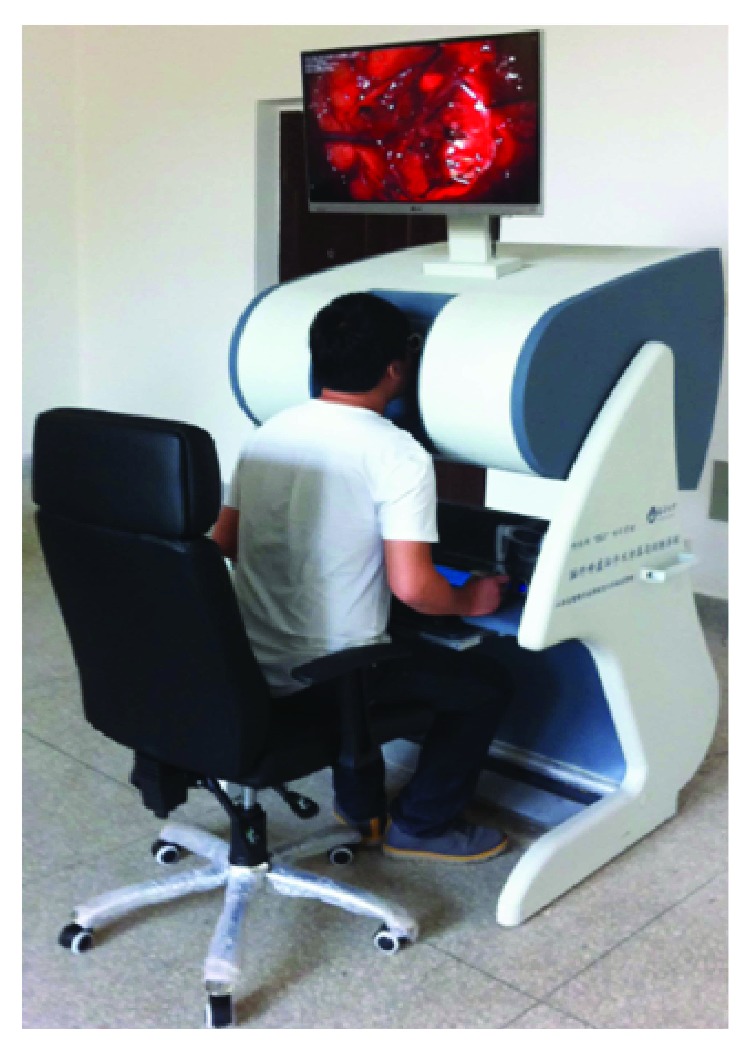
The neurosurgery virtual simulation system.

**Figure 18:**
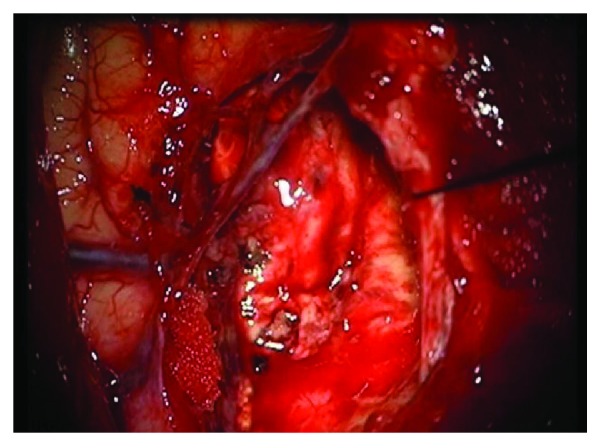
Virtual neurosurgery.

**Figure 19:**
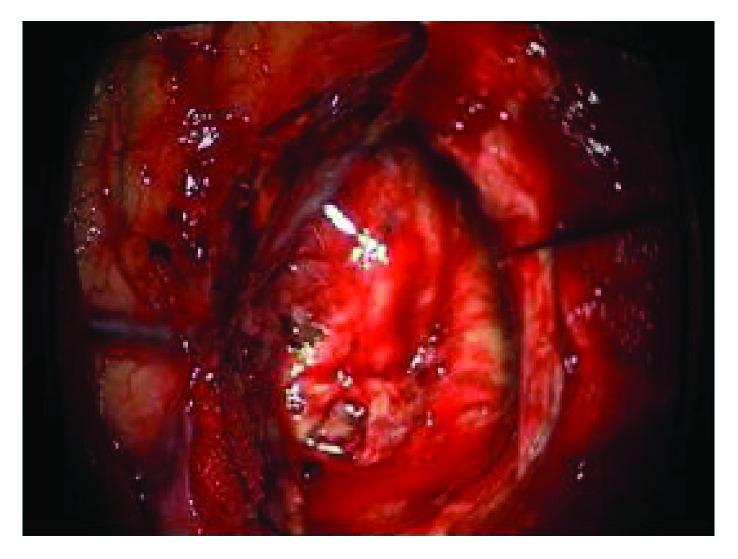
Real neurosurgery.

**Figure 20:**
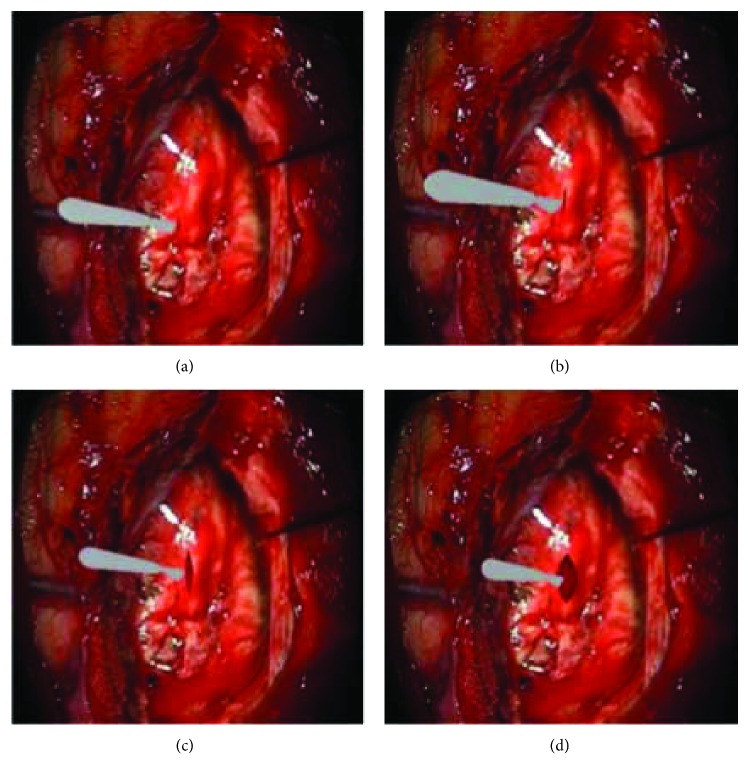
Simulation of the cutting of the cerebroma. (a) Cutting preparation, (b) starting cutting, (c) small incision, and (d) large incision.

To further validate the proposed approach, our coupled model was implemented into the neurosurgery virtual simulation system. This system can simulate the common cerebroma cutting process in neurosurgery. The system consists of several parts: external 3D display, inner 3D display, 3D glasses, graphics workstation, two haptics, and an operating platform (see Figure 17). And the system is built on a graphics workstation with an Intel Xeon E5-2630 (v3) CPU, an NVIDIA Quadro K5200. To simulate a real surgical scene, the texture used in the rendering subsystem is extracted from a real surgery video. The virtual human cerebroma tissue (length: 50 mm, width: 40 mm, and height: 30 mm) in Figure 18 consists of 2601 surface triangles and 7086 internal point elements. The surrounding blood vessels and other tissues of the cerebroma are the real surgery scene screenshot.  Figure 19 is a real surgical scene which shows a cerebroma in the human brain. It can be observed in Figure 18, the reconstruction of virtual model is quite similar to the real operation. In Figure 20, we show various stages of the partial excision of cerebroma simulation (final size of the incision—length: 8 mm, width: 5 mm, and height: 4 mm). Along the surgery, the scalpel penetrates into the soft tissue and the cerebroma is gradually cut. As can be seen in Figure 20(d), the cutting surface of the incision is smooth and the shape of the incision is consistent with the real incision. Therefore, the coupled model provided satisfactory visual effects. In addition, users can interact with the virtual system via force feedback device, which provides the realistic force feedback to the hands of users, making users to get more satisfaction of immersion and interactivity.

Finally, to test the computational efficiency, we have halved models of the liver, shoulder, and cerebroma with varying surface mesh and internal point element sampling densities, following the proposed coupled cutting algorithm. The results of the developed method are listed in Table 1. As shown in Table 1, the total cutting time for the three models ranged from 2.335 ms to 27.53 ms. And the visual refresh rate ranged from 36 Hz to 428 Hz. In general, a visual refresh rate of 30 Hz can meet real-time display. Therefore, due to the level set method which greatly improves the rate of computations, the coupled model provides the means for the user's real-time interaction.

## 7. Conclusion

In this paper, we proposed a new coupled soft tissue cutting simulation model which not only takes full advantage of the two types of models, that is, mesh and meshless models, but also avoids their drawbacks. The proposed approach utilizes the mesh method to describe the surface of soft tissue and uses the meshless point elements to describe internal soft tissue. The introduced virtual points combine surface mesh and internal point elements together and the internal deformation synchronizes with the surface cutting incision. The surface shape of the incision described by the Bezier curve is smooth and realistic. The deformation displacement of the internal point elements simulated by displacement vector gradient algorithm can well characterize the shape deformation of internal soft tissue. Moreover, by modifying the force feedback with an oscillating function, the simulation is more realistic.

To improve the operation efficiency, we use level set method to track the variation of the cutting plane. The internal point elements of the model are divided into two regions according to the position of the cutting plane, that is, the cutting-affected region and cutting-unaffected region. The point elements that are in the unaffected region do not need to update, and our method only needs to calculate the displacements of the point elements in the cutting-affected region.

In addition, the convergence and stability of our model are discussed and theoretically proven. The real biomechanical experimental results show that the force calculated from the proposed model has a good consistency with the experimental measurement. And the maximum relative error is less than 6.2%. Convergence studies on the biomechanical testing using RMSE show that the proposed method possesses a satisfactory rate of convergence.

Finally, the proposed approach has been successfully applied to a neurosurgery virtual simulation system. Since our model can offer a very high level of visual realism and accurate interactive force feedback, it makes the users obtain excellent experience of immersion and interactivity. In future work, we plan to improve the cutting model with bleeding and suturing to enhance the reality of the surgical simulation system.

## Figures and Tables

**Table 1:** Computation time of the cutting simulation for different model scales.

Model scale	Number of surface meshes	Number of internal points	Surface cutting time (ms)	Internal cutting time (ms)
Liver	508	1526	0.125	2.21
Shoulder	1842	4812	1.03	9.08
Cerebroma	2601	7086	6.86	20.67
